# Effects of glucose and trehalose on tris-citric acid-egg yolk-fructose diluents for semen cryopreservation in goat

**DOI:** 10.5455/javar.2023.j666

**Published:** 2023-06-30

**Authors:** Md. Mostofa Kamal, Md. Emtiaj Alam, Sunny Kumar Das, Most. Shorifa Yeasmin, Soshe Ahmed, Mst. Afroza Rahman, Dipak Kumar Das, Md. Royhan Gofur, Md. Abdul Masum

**Affiliations:** 1 Department of Veterinary and Animal Sciences, Faculty of Veterinary and Animal Sciences, Rajshahi University, Rajshahi, Bangladesh; 2 Department of Pharmacy, Faculty of Sciences and Engineering, East West University, Dhaka, Bangladesh; 3 Department of Anatomy, Histology and Physiology, Faculty of Animal Science and Veterinary Medicine, Sher-e-Bangla Agricultural University, Dhaka, Bangladesh

**Keywords:** Buck, cryopreservation, glucose, sperm, sperm parameter, trehalose

## Abstract

**Objectives:**

This study aimed to examine the impacts of the wide range of concentrations of glucose and trehalose on the tris-citric acid-egg yolk-fructose (TCEF) extenders for cryopreservation of goat semen.

**Materials and Methods:**

The sperm sample was pooled, washed, and diluted in control (TCEF without glucose and trehalose), TCEF + glucose (75, 150, 450, and 900 mm), and TCEF + trehalose (75, 150, 450, and 900 mm). After equilibrations, the semen straws were frozen under LN2 in the LN2 tank. After LN2 storage, the straws were thawed at 37°C for 30 seconds. The sperm parameters of all study groups were checked after equilibration and freezing.

**Results:**

After equilibration, the progressive motility (PM), total motility (TM), and viability of sperm in G-75, G-150, G-450, T-75, T-150, and T-450 were not significantly different (*p* < 0.05) from those in control. After cryopreservation and thawing, the PM, TM, and plasma membrane integrity (PMI) of T-150 were significantly higher (*p* < 0.05) than in control, G-75, G-900, T-75, and T-900. The viability of sperm in T-150 was substantially higher (*p* < 0.05) than in the control, whereas there was no significant difference among the control, G-75, G-900, T-75, and T-900. However, the acrosome integrity (AI) of sperm in G-900 was significantly decreased (*p* < 0.05) compared to the control, G-75, G-150, G-450, T-75, T-150, and T-450.

**Conclusion:**

According to the findings, the supplementation of 150 mm trehalose in the TCEF diluent was more efficient for sperm cryopreservation in the buck as reflected by PM, TM, viability, PMI, and AI.

## Introduction

Sperm cryopreservation technology, which helps to preserve sperm over long periods, is crucial for advancing artificial insemination (AI), accelerating breeding, and preserving genetic resources. However, during the freezing steps, semen motility, membrane integrity, and fertilizing ability are decreased due to cold shock, the production of reactive oxygen species, and osmotic stress [1–5]. Numerous studies have revealed that adding sugar to an extender positively impacts the motility, vitality, and plasma membrane integrity (PMI) of mammalian semen after cryopreservation [6–9]. Various studies used fructose or glucose in a tris-citric acid diluent to freeze mammalian sperm [10–14].

Fructose is a key ingredient for glycolysis in buck seminal plasma, so it’s logical to add this sugar to the medium [15]. In contrast, glucose is a notable substrate for sperm metabolism and is necessary for the energy supply for the sperm to function normally [16,17]. Fructose and glucose can cross the plasma membrane of sperm due to their low molecular weight.

Furthermore, it has been previously stated that in rams, monosaccharides (fructose and glucose) exhibit superior cryoprotective qualities than disaccharides (lactose, sucrose, or trehalose) [18]. Another study, however, indicated that combining fructose with trehalose increased the viable sperm in rams [19]. Unlike the simple sugars glucose and fructose, trehalose has been added to buck semen freezing extenders. This disaccharide is typically employed as a cryoprotectant. Trehalose can be integrated into the plasma membrane to reduce the amount of dehydration of sperm and, as a result, decrease the physical damage brought on by variations in cell volume caused by freezing and thawing [20]. It also increases membrane fluidity by reorganizing proteins and phospholipids and suppressing the harmful effects of the membrane lipid phase transition. Less intracellular ice formation generally reduces cellular damage and increases survivability after cryopreservation [21,22].

Although certain sugar-containing diluents, including glucose-fructose-raffinose yolk and sodium citrate-glucose yolk, have been examined for their possible use in freezing goat sperm [23-24], however, no specific additives in semen dilution have yet been established for the cryopreservation of goat semen. Even though diverse mammals frequently employ glucose and trehalose for semen cryopreservation [19,25–27]. Furthermore, some studies have used various concentrations of glucose and trehalose in multiple types of diluents for buck semen cryopreservation [21,28–30], and the results have varied among these studies.

Aboagla and Terada [21] used up to 375 mm of trehalose with 4% glycerol and found this concentration provided the best result in the Shiba goat. Another report observed that 50-150 mm trehalose supplementation with 5% glycerol [28] did not enhance semen quality in Black Bengal buck after freezing. Li et al. [30] used 0, 28, 56, 84, and 112 mm glucose and found 56 mm could improve semen motility, the percentage of the intact acrosome, and the plasma membrane in frozen-thawed Cashmere goat semen. Furthermore, Naing et al. [29] reported that sperm forward motility in post-thaw goat semen was improved by glucose as opposed to trehalose or sucrose supplementation in Boer goats.

So, it is indicated that the sugar effect is varied among the studies, species, and the cultural environment. In addition, to the best of our knowledge, no report compares various amounts of glucose and trehalose at the broad range level of the tris-citric acid-egg yolk-fructose (TCEF) diluents for buck semen cryopreservation.

Therefore, the purpose of this study was to examine the impacts of the wide concentration range of glucose and trehalose to identify more effective sugars and their optimal concentrations in the TCEF diluents for long-term freezing of the buck spermatozoa.

## Materials and Methods 

### Ethical statement

This work was approved (110(16)/320/IAMEBBC/IBSc) by the Institutional Animal, Medical Ethics, Biosafety, and Biosecurity Committee (IAMEBBC) of Rajshahi University in Bangladesh.

### Research period and animal selection

The experiment was performed from February to July 2022. For this experiment, three adult beetal bucks were used. They weighed more than 50 kg and ranged in age from 20 to 36 months. The bucks were housed and cared for at the veterinary clinics and AI center, Naricalbaria, run by the Veterinary and Animal Sciences Faculty, University of Rajshahi. A daily diet of 600 gm of concentrate, 450 gm of rice straw, 2.5 kg of Napier grass, unlimited amounts of fresh water, and sunlight during the day was provided to the experimental bucks.

### Semen extenders

At first, a [tris-citric-acid-fructose(TCF)] solution was made using our prior study’s [2] methodology, with a few modifications: tris-3.41 gm (Himedia Lab. Pvt., Ltd. Mumbai 400086, India.,), citric acid-1.61 gm (Sigma-Aldrich Corp., St. Louis, MO 63103, USA), fructose-0.81 gm (Himedia Lab. Pvt.,), with deionized water (RSL Lab. Ltd., Bangkok 10330, Thailand). After that, the following extenders were created individually without or with adding D(+)- glucose (Wako PureChe., Corp., Osaka 540-8605, Japan) and trehalose (Himedia Lab. Pvt.,) to the TCF solution: control without glucose (G) and trehalose (T), G-75 mm, G-150 mm, G-450 mm G-900 mm glucose and T-75 mm, T-150 mm, T-450 mm, T-900 mm trehalose. Gentamicin (250 μg/ml) and egg yolk 16% (*v*:*v*) should be introduced to each extender group after proper mixing (50°C to 70°C and 30 to 200 rpm for 15 min) and cooling (4°C). The pH should be maintained at 7.00; each extender should make up to 100 ml by adding deionized water. The entire extender groups were thoroughly mixed again using a magnetic stirrer with a gradually increasing rotation setting (30–200 rpm) and without temperature. Extenders were separated from their supernatant after being centrifuged at 3,000 rpm at room temperature for 15 min. For cryopreserving semen, each set of extenders was split into Part-1, which included no glycerol, and Part-2, which had 14% glycerol, leading to a final concentration of 7% glycerol.

### Semen collection

The semen collection and selection parameters were carried out, according to Kamal et al. [2], with some modifications. To summarize, a doe stimulated the buck, and semen was collected twice a week (at around 5-day intervals) using an artificial vagina. A total of 10 aliquots (3-4/goat) were used in this study. Following sperm collection, the sperm with TCF-washing media containing falcon tubes were placed in a 37°C water bath. We prepared a TCF washing solution with tris-3.41 gm, citric acid-1.61 gm, and fructose-0.81 gm in 100 ml deionized water, pH 7.0. The semen parameters were assessed, including volume (ml), color, mass movement (0–4), semen concentration (*n *× 10^9^), sperm progressive motility (SPM), sperm total motility (STM), sperm viability (SV), sperm plasma membrane integrity (SPMI) and sperm acrosome integrity (SAI). The two researchers evaluated each sperm parameter visually and separately throughout the investigation to increase the reliability of the findings. Ejaculates with a volume of ≥0.65 ml, color creamy white, mass movement ≥3.20, concentration ≥2 × 10^9^ sperm/ml, SM ≥ 80%, SV ≥80%, SPMI ≥70%, and SAI ≥80% were selected for this research.

### Semen evaluations

#### Sperm progressive motility and total motility

The SPM and STM were examined by our prior investigation [2], with certain modifications. In brief, the SPM and STM were assessed after 10 μl of semen were diluted with the prewarmed (at 37°C) washing solution. Then an aliquot (10 μl) of semen was taken on the prewarmed clean glass slide (at 37°C) and, after that, covered with a prewarmed coverslip (18 × 18 mm). All slides were observed at 400× under bright field microscopy, and 200 sperm were analyzed from at least three separate fields. Following the recommendations of the WHO [31], SPM and STM were counted.

### Sperm viability

**Figure 1.**
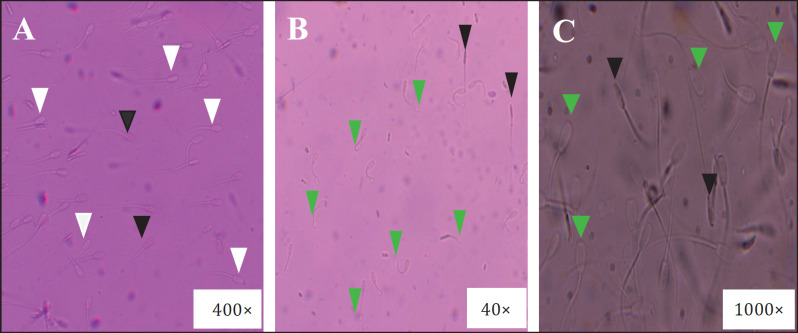
Evaluation of buck sperm using different tests. A. semen viability check by eosin-nigrosine staining; the white arrow indicates live, and the black arrow indicates dead sperm. B. SPMI check by HOST with staining; the green arrow indicates intact plasma membrane, and the black arrow indicates the damaged plasma membrane. C. Semen acrosomal integrity check by 1% formal citrate solution; the green arrow indicates the intact acrosome and the black arrow indicates the damaged acrosome.

The SV of semen was checked by the eosin-nigrosin staining technique according to the previous reports [2,32] with some modifications. Briefly, distilled water was used to dissolve 1% eosin (Sigma-Aldrich Corp.), 5% nigrosin (Himedia Lab. Pvt., Ltd.), and 3% trisodium citrate dihydrate (Himedia Lab. Pvt., Ltd). The mixture was gently heated while constantly stirring; boiling was not used. Once dissolved, let the solution cool, and then store it in a 15-ml falcon tube covered with aluminum foil at room temperature. The 10 μl of sperm and 10 μl of this stain were properly mixed in an Eppendorf tube and allowed to wait for at least 30 seconds. Take 10 μl of stained sperm to a slide, cover it with a coverslip, and count the sperm after it has settled. Two hundred sperm from at least three different fields were examined using a bright-field microscope (400×), and the proportions of live (unstained/white heads) and dead (pink/redheads) sperm were noted (Fig. 1A).

### Sperm plasma membrane integrity

The PMI of the semen was assessed using the hypoosmotic swelling test (HOST). According to Kamal et al. [2], Jeyendran et al. [33], and Revell and Mrode [34], the HOST solution was made. The functional integrity of sperm was examined by the HOST following staining under a light microscope [2]. After that, we placed a prewarmed (at 37°C) cover slip over the sperm aliquots, and the percentage of sperm exhibiting coiling and swelling of the tail was counted under a light microscope at 400× (Fig. 1B). There were 200 sperm examined from at least three separate fields.

### Sperm acrosomal integrity

According to Khan and Ijaj’s [32] analysis, the integrity of the sperm acrosomes was evaluated. Briefly, 2.9% (*w*/*v*) trisodium citrate dihydrate (Himedia Lab. Pvt., Ltd.) and 1% (*v*/*v*) commercial formaldehyde 37% (Merck, Germany) were dissolved in distilled water to create a 1% solution of formal citrate. A 25 μl solution of 1% formal citrate was used to fix 250 μl of semen. Transfer 10 μl of sperm onto a slide, smear it, and then cover it with a cover slip. 100 sperm were evaluated for the existence of a normal apical ridge using a light microscope at 1,000× magnification. Acrosome-containing sperm were considered normal if they had a typical, smooth, crescentic acrosome region (Fig. 1C).

### Experimental design

This study aimed to assess the various glucose and trehalose concentrations in high-quality buck sperm. The pooled ejaculate was gently pipetted and separated into nine equal portions in nine distinct, clearly labeled falcon tubes with the washing solution. The tubes were labeled with control (0%, G, and T), G-75, G-150, G-450, G-900, T-75, T-150, T-450 and T-900. Then add additional tris-based washing media to every nine tubes for washing semen. The collected ejaculates were centrifuged to wash them for 10 min (at 1,000 rpm). After centrifugation, the supernatant was aspirated to extract the semen plasma from each tube. Each marked tube of sperm was mixed with the corresponding group in the first part of the extender without glycerol and kept at 4°C for 3 h. The next two experiments were then conducted.

In experiment 1, the effects of various glucose and trehalose concentrations on the spermatozoa quality following equilibration were examined. An aliquot (20 μl) of sperm was pipetted gently before being added to the prewarmed tris-based washing media and kept at 37°C in the water bath for 5 min. All nine extender groups’ SPM, STM, and SV percentages were checked. Experiment 2 examined the impact of various quantities of glucose- and trehalose-containing extenders on the sperm parameter after freezing-thawing of sperm. To obtain the final concentration of 2.5 × 10^7^ spermatozoa/ml with a final glycerol concentration of 7%, the second portion of each extender, which contains 14% glycerol, was added to the solution following the equilibration of sperm. The diluted semen samples were put into 0.25 ml straws (Instruments de Médecine Vétérinaire Technologies, France). The straws were sealed, held above 4 cm of liquid nitrogen (LN2) in a horizontal position for 15 min, and then placed into a tank of LN2 for storage. The straws containing cryopreserved sperm were thawed in a water bath (at 37°C) for 30 sec after at least 1 day of storage. The percentages of SPM, STM, SV, SPMI, and SAI were assessed for each of the extenders’ groups.

## Results

### Effect of different concentrations of glucose and trehalose on SPM, STM, and SV of buck semen after equilibration

The findings of experiment 1 are mentioned in Table 1. After equilibration, the PM of G-75, G-150, G-450, T-75, T-150, and T-450 were not significantly different from that of the control. Whereas, the G-900 and T-900 were significantly decreased (*p *< 0.05) than the control, G-75, G-150, T-75, T-150 and T-450. However, there were no statistically significant differences between G-900 and T-900 or between G-450 and G-900.

The TM of G-75, G-150, G-450, T-75, T-150, and T-450 showed no significant differences from the control. However, the G-900 and T-900 were significantly decreased (*p *< 0.05) than the control, G-75, G-150, T-75 and T-150. Furthermore, there were no significant differences between G-900 and T-900 and also among the G-450, G-900, and T-450.

The viability of G-75, G-150, G-450, T-75, T-150, and T-450 were not significantly different from than control. However, the G-900 and T-900 were substantially lower (*p *< 0.05) than the control and other treatments. In addition, there were no significant differences between G-900 and T-900.

### Effect of different concentrations of glucose and trehalose on SPM, STM, SV, SPMI, and SAI of buck semen after cryopreservation

The findings of Experiment 2 are mentioned in Table 2. After buck sperm was frozen in LN2, the PM of T-150 provided the highest percentages and was significantly higher (*p* < 0.05) than the control, G-75, G-900, T-75, and T-900. At the same time, there were no significant differences among the G-450, T-150, and T-450.

The TM of sperm in T-150 was significantly increased (*p* < 0.05) compared to the control, G-75, G-900, T-75, and T-900. In contrast, there were no significant differences among the G-150, G-450, T-150, and T-450.

The viability of sperm in T-150 was significantly higher (*p* < 0.05) than in the control, whereas there were no significant difference among the control, G-75, G-900, T-75, and T-900. In addition, there were also no significant differences among the G-75, G-150, G-450, G-900, T-75, T-150, T-450, and T-900.

The PMI of sperm in T-150 was significantly higher (*p* < 0.05) than that in control, G-75, G-900, T-75, and T-900. However, no significant differences existed among the control, G-450, and T-450. There were also no significant differences among G-150, G-450, T-150, and T-450.

The acrosome integrity (AI) of sperm in G-900 was significantly decreased than the control, G-75, G-150, G-450, T-75, T-150, and T-450. However, there were no significant differences between G-900 and T-900.

**Table 1.** Seminal parameter status of the bucks after being equilibrated without and with various concentrations of glucose and trehalose in TCEF based extenders.

Sugar	Concentration (mm)	Characteristics of spermatozoa (%)		
		PM	TM	Viability
Control (without G and T)		62.639 ± 1.688^a^	67.290 ± 1.421^a^	68.606 ± 1.261^ab^
	G-75	62.481 ± 1.808^a^	67.114 ± 2.000^a^	69.602 ± 1.861^b^
Glucose (G)	G-150	61.470 ± 3.086^a^	66.176 ± 3.164^a^	69.820 ± 1.782^b^
	G-450	58.188 ± 2.741^ab^	63.255 ± 2.413^ab^	64.170 ± 2.498^a^
	G-900	53.896 ± 1.244^bc^	58.797 ± 1.485^bc^	58.276 ± 1.213^c^
	T-75	60.173 ± 0.971^a^	64.903 ± 1.020^a^	66.254 ± 0.995^ab^
Trehalose (T)	T-150	60.349 ± 1.275^a^	65.010 ± 1.211^a^	66.288 ± 1.414^ab^
	T-450	60.883 ± 2.838^a^	64.223 ± 2.735^ab^	65.338 ± 1.863^ab^
	T-900	50.552 ± 1.263^c^	57.559 ± 1.609^c^	58.934 ± 0.718^c^

Values are mean ± SE. ^a-c^ Different superscripts indicate statistical differences (*p *< 0.05) within a column.

**Table 2.** Seminal parameter status of the bucks after being cryopreservation without and with various concentrations of glucose and trehalose in TCEF based extenders.

Sugar	Concentration (mm)	Characteristics of spermatozoa (%)				
		PM	TM	Viability	HOST positive following stained	AI
Control (without G and T)		4.877 ± 2.354^ab^	6.335 ± 3.036^ab^	38.387 ± 2.209^a^	5.612 ± 2.614^abd^	53.885 ± 2.236^a^
	G-75	1.185 ± 0.790^a^	2.126 ± 1.557^a^	42.804 ± 3.690^ab^	2.159 ± 1.439^b^	53.095 ± 1.542^a^
Glucose (G)	G-150	8.994 ± 2.122^bc^	10.621 ± 2.423^bc^	45.739 ± 2.805^b^	10.275 ± 2.400^cd^	53.903 ± 2.640^a^
	G-450	9.106 ± 3.722^bd^	9.927 ± 4.061^bc^	45.533 ± 0.847^b^	10.335 ± 3.107^ac^	53.152 ± 0.639^a^
	G-900	0.000 ± 0.000^a^	0.000 ± 0.000^a^	41.250 ± 2.000^ab^	0.405 ± 0.405^b^	47.419 ± 1.632^b^
	T-75	1.663 ± 0.887^a^	3.224 ± 1.664^ab^	43.066 ± 2.315^ab^	2.771 ± 1.414^b^	53.862 ± 2.026^a^
Trehalose (T)	T-150	15.341 ± 3.084^cd^	17.012 ± 3.660^c^	47.339 ± 2.882^b^	13.210 ± 4.254^c^	53.609 ± 1.962^a^
	T-450	9.143 ± 3.742^bd^	9.778 ± 3.999^bc^	45.421 ± 0.889^b^	11.219 ± 2.583^ac^	53.766 ± 0.548^a^
	T-900	0. 000 ± 0.000^a^	0.000 ± 0.000^a^	41.310 ± 2.428^ab^	1.013 ± 0.692^b^	51.125 ± 1.577^ab^

Values are mean ± SE. ^a-d^ Different superscripts indicate statistical differences (*p* < 0.05) within a column.

### Statistical analysis

All experiments were subjected to an arcsine transformation. A one-way ANOVA and the Least Significant Difference test were employed to analyze statistical differences in sperm parameters (SPSS-26.0, a statistical package, SPSS Inc., Chicago, IL). In each case, *p* < 0.05 was considered statistically significant.

## Discussion

The cryopreservation process is widely used for the long-term storage of semen; however, one should not neglect its adverse effects, such as motility issues, a decline in viability rate, and other sperm parameter defects [2,35–36]. At the same time, exogenous sugar in a semen extender is primarily used as an energy source for sperm and plays an essential role in protecting them from the harmful effects of cryopreservation [16,37]. In this study, various concentrations of glucose and trehalose in TCEF diluents were compared to the efficiency of goat sperm after equilibration and cryopreservation. The present study’s findings showed a beneficial effect of trehalose to a TCEF extender on goat semen quality after freezing.

The results of this study revealed improved sperm PM, TM, viability, and PMI, while AI was maintained, especially with trehalose-150 mm (T-150) after cryopreservation. In agreement with our findings, the previous report showed that the hypertonic diluents provided the highest motility and ultrastructure protection of semen after cryopreservation [6,19,38–40]. Trehalose provides the improved quality of semen in buck due to minimizing the injury caused by ice crystallization. At the same time, trehalose acts as a non-permeable sugar creating hypertonic media that decreases the freezable water at the intracellular level [41]. Furthermore, other studies mentioned that the cryodamage of sperm was reduced due to the interaction of this sugar with the phospholipids in the plasma membrane, which enhanced membrane fluidity and resulted in more excellent resistance of sperm to freeze-thaw damage [21,42]. In addition, trehalose has indirect antioxidant activity by boosting glutathione levels and decreasing the level of lipid peroxide [43], which improves post-thaw sperm quality. It also has a cryoprotective impact on the acrosome and mitochondria, which generate energy from intracellular adenosine triphosphate (ATP) stores, resulting in better semen motility after cryopreservation [42].

Hence, it is indicated that trehalose has a favorable effect on improving the quality of semen after freezing and thawing. Although the proportion of results decreased, there were no significant differences of the glucose-150 mm (G-150) and glucose-450mm (G-450) than T-150 after freezing and thawing. In agreement with the findings, Molinia et al. [38] showed in ram that the motility of frozen-thawed sperm is higher in the presence of trehalose than in the presence of glucose when glycerol is absent in the diluents; however, no changes were seen when glycerol was present in the diluents. According to one report, disaccharides (sucrose, trehalose) are more cryoprotective than monosaccharides (glucose, galactose, or fructose) in boar [44]; in contrast, a different study found that glucose supplementation significantly increased (*p *< 0.05) the forward motility of post-freeze goat semen compared to trehalose or sucrose supplementation [29]. Thus, the impact of glucose and trehalose on sperm freezing appears to vary by species and possibly by the different techniques applied.

Furthermore, the current study found that adding more trehalose-450 mm (T-450) did not increase the sperm parameters, and a high level of glucose-900 mm (G-900), and trehalose-900 mm (T-900) caused the sperm parameters like SPM, STM, and SPMI to decrease significantly compared to T-150 after cryopreservation. A previous study found that the ram sperm may survive hyperosmotic diluents at sugar concentrations between 50 and 100 mm for better post-thaw semen quality [45]. According to Zhu et al. [46], during the boar sperm incubation process, ATP production from the glycolysis pathway was reduced when the glucose level in the incubation medium was increased, implying that boar sperm can change the metabolic way. They also found in another study that high-glucose media might stimulate sperm with increased glycolysis, resulting in a disturbance of sperm cellular homeostasis and lower semen quality and function in boars [25].

External osmolarity affects sperm motility [19,47–50], where high concentrations of sugar are crucial for the high osmolality of the extender, which is harmful to the semen cells. This is consistent with the findings of a previous study, where 200 and 400 mOsm trehalose had a deleterious impact on post-thaw motility and functional integrity in ram [19]. Furthermore, using hypertonic extenders (tris-citrate modified solution) plus 76 g/l trehalose significantly reduced semen functional membrane damage in rams [43]. The exact mechanism underlying the detrimental effects of elevated trehalose concentrations is unclear; however, higher concentrations may make the medium hypertonic, raising external pressure on semen and modifying cell architecture via membrane protein denaturation, lipid phase transitions, and decreased membrane fluidity [51]. Higher trehalose concentrations enhanced sperm viscosity, resulting in reduced semen motility due to reduced sperm flagella movement, partially regained by diluting the freezing solution [52]. The increase in diluted osmolarity, which leads to a decline in individual PM, TM, viability, and PMI, may be the root of the adverse effects of higher sugar content in this study.

When we compare the other report on the buck, Aboagla and Terada [21] obtained 375 mm trehalose in 4% glycerol-containing diluents, which had a good effect on the Shiba goat. However, other reports found that adding 25-75 mm [53] and 50-150 mm trehalose with 5% glycerol [28] did not enhance the semen quality in Angora and Black Bengal buck, respectively. Moreover, the overall sperm motility decreased compared to other studies [21]. Still, we found that 150 mm trehalose in 7% glycerol enhanced the PM, TM, viability, and PMI after freezing. This suggests that there may be a crucial difference between species regarding the optimal trehalose and glycerol concentration, and different factors like extender composition, washing/non-washing of sperm, and processing temperatures can influence sperm quality after freezing. The findings of this study suggested that the trehalose used in the TCEF-containing extender in the presence of glycerol can significantly improve the efficiency of goat sperm cryopreservation. However, glucose has a potential effect.

In this study, although trehalose had a good impact on the quality of buck sperm after cryopreservation, post-thaw motility, and PMI were suboptimal for AI [54]. Therefore, further research may be implemented using various ratios of trehalose and glycerol to maximize the survivability of frozen sperm for AI of does.

## Conclusion

In conclusion, adding 150 mm T in the TCEF diluents was found to be more efficient for goat sperm cryopreservation, as reflected by PM, TM, SV, PMI, and AI. Furthermore, our findings support the benefits of trehalose in protecting buck sperm during cryopreservation.

## List of Abbreviations

AI, Acrosome integrity; AI, Artificial insemination; ATP, Adenosine triphosphate; G, Glucose; IAMEBBC; Institutional Animal, Medical Ethics, Biosafety, and Biosecurity Committee; IMV, Instruments de Médecine Vétérinaire; Kg, Kilogram; LN2, Liquid nitrogen; LSD, least significant difference; TCEF, Tris-citric acid-egg yolk-fructose; TM, Total motility; T, Trehalose; TCF, tris-citric-acid-fructose; PM, Progressive motility; PMI, Plasma membrane integrity; SPM, Sperm progressive motility; STM, Sperm total motility; SV, Sperm viability; SPMI, Sperm plasma membrane integrity; SAI, Sperm acrosome integrity
